# Artificial Saliva: Challenges and Future Perspectives for the Treatment of Xerostomia

**DOI:** 10.3390/ijms20133199

**Published:** 2019-06-29

**Authors:** Dawid Łysik, Katarzyna Niemirowicz-Laskowska, Robert Bucki, Grażyna Tokajuk, Joanna Mystkowska

**Affiliations:** 1 Department of Materials Engineering and Production, Bialystok University of Technology, Wiejska 45C, 15-351 Bialystok, Poland; 2 Department of Experimental Pharmacology, Medical University of Bialystok, Szpitalna 37, 15-295 Bialystok, Poland; 3 Department of Microbiological and Nanobiomedical Engineering, Medical University of Bialystok, Mickiewicza 2C, 15-222 Bialystok, Poland; 4 Department of Integrated Dentistry, Medical University of Bialystok, M. Sklodowskiej-Curie 24a, 15-276 Bialystok, Poland

**Keywords:** artificial saliva, xerostomia, rheology

## Abstract

The chronic sensation of a dry mouth is a disease condition called xerostomia and affects a large part of the population. Xerostomia is associated with decreased secretion, or more often, qualitative changes in saliva proteins and immunoglobulin concentrations that develop as a result of salivary gland dysfunction. Several reasons causing dry mouth were described, and usually, they include taking medications, diseases or radiotherapy. In some situations, when it is difficult to use salivary stimulants or salivary gland damage is irreversible, the only option might seem to be saliva substitutes. The paper presents the most important aspects considering saliva preparations. The rheological and lubricating properties and the reconstruction of the complex saliva structure has been the main purpose of research. The biological properties of saliva preparations were also widely discussed. As part of the work, the antimicrobial effect of three commercial saliva preparations was tested. Finally, inadequate antimicrobial properties against the strains isolated from the oral cavity were demonstrated. The development of salivary substitutes, in particular, the improvement of antimicrobial properties, can be achieved using nanotechnology, including drug delivery systems containing nanocarriers.

## 1. Introduction

Most people who have been upset, anxious or under stress have experienced a dry mouth. This is a subjective feeling of reduced secretion of saliva in the mouth, associated not only with its amount but rather regarding quantitative and qualitative changes in saliva composition. The feeling of dry and cracked lips, sticky and viscous saliva, altered taste and smell, difficulty talking, problems with chewing, tooth caries and their increased erosion, heartburn and reflux exacerbation, oesophagitis, burning tongue, festering and irritating mucous membrane infections are the consequences of salivary gland dysfunction [1]. 

Living with reduced saliva secretion is not only difficult but also leads to serious health problems such as xerostomia. In such situations, therapeutic methods for stimulation of saliva secretion are used. However, in some cases, salivary gland damage requires continuous use of saliva substitutes [2,3,4]. The main purpose of this review was an analysis of the most important aspects concerning saliva preparations due to its microbial, rheological and lubrication properties.

## 2. Xerostomia: Etiology of Salivary Glands Dysfunctions

**Figure 1:**
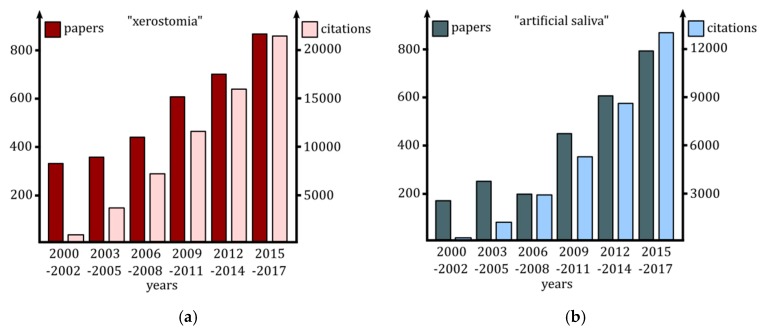
A number of articles and citations regarding the terms: (**a**) “xerostomia”, (**b**) “artificial saliva” according to the Web of Science database.

The chronic sensation of dry mouth leads to a disease entity called xerostomia. The number of undesirable factors affecting salivary glands, people suffering from stress, exposed to many diseases, and aging can make xerostomia a global problem. In prospective population studies [5] (*n* = 2942, adults aged 20–59), it was shown that regular xerostomia symptoms concern about 3.8%, while irregular, 12.2% of the population. In studies of older people [6] (*n* = 600, over 70 years of age) in Japan, the hyposalivation problem was observed in 37.3% of patients (27.8% in men and 47.3% in women). In other studies, Cardoso et al. [7] found that 45.5% of disease-free oropharyngeal cancer survivors (*n* = 906) reported problems with dry mouth. Rising interest in xerostomia and methods of its treatment, especially using artificial saliva, is currently observed. This is clearly visible in the bibliometric data (Figure 1), according to the Web of Science database. The term “xerostomia” in years 2000–2018 was found in 3671 publications, which were quoted more than 70,000 times, while the term “artificial saliva” related to 2757 publications, which were cited about 37,000 times.

Interestingly, patients complaining of dry mouth sometimes do not show any objective symptoms of hyposalivation. The diagnosis of xerostomia requires a detailed medical history, which includes a detailed description of the symptoms (patients most often complain of dry mouth, difficulty in swallowing and speaking, do not tolerate acute and acidic taste), diseases and the use of medicines. Nevertheless, the measurement of salivary flow is the basis for the diagnosis of xerostomia. However, it can be a problem to determine the amount of saliva that is indicative of the dysfunction of the salivary glands [8].

The term saliva, by default, refers to the terms "whole saliva" or "mixed saliva", which are used to describe the combined fluids present in the oral cavity. Measurement of its quantity is a good method to determine the degree of dryness of the mouth, while the measurement of salivary secretion from specific salivary gland allows determination of its individual efficiency [1].

Saliva can be classified as resting (unstimulated) and stimulated. A main protective function of oral tissues is ascribed to resting saliva since it is present in the oral cavity for about 14 h a day. Stimulated saliva is secreted in the mouth for about 2 h a day, and its role is mainly related to alimentary functions. The average daily flow of whole saliva varies in health between 1 and 1.5 L. The unstimulated saliva flow rate is in the range of 0.3–0.7 mL per minute. Mechanical, chemical or psychoneurological stimulation increases the flow rate to 1.5–2 mL per minute [1]. Hyposalivation is observed when resting salivary flow rate decreases below 0.1 mL per minute and stimulated saliva below 0.5–0.7 mL per minute [1,9,10]. The salivary flow rate is usually measured 5 min after waking up or 2 h after the last meal. Unstimulated saliva flow is measured in a sitting position for 15 min, collecting saliva from the lower lip. Saliva can also be collected with cotton rolls, placed near the salivary glands (the differences in the weight of the rolls before and after the test should be taken into account). Another way is to use special, calibrated absorbent straps placed on the floor of the mouth. Stimulated saliva is collected after chewing gum or paraffin wax by the patient, or stimulation with 2% citric acid solution (placed on the sides of the tongue) [11]. The secretion of the parotid gland is usually collected by means of a suction device and a cup (Lashley or Carlson-Crittenden cup) placed over the Stensen duct [12]. In a similar way, the flow from the submandibular gland can be examined by isolating the Wharton’s duct [13]. There are also flow measurement systems from smaller salivary glands, including, for example, the use of micropipettes and filter papers [14].

There are many factors that can cause xerostomia [15]. The main reason is taking medication, especially from the anticholinergic [16,17,18], sympathomimetic [19,20,21,22] and antihypertensive [23] groups. Some opioids, benzodiazepines [24,25] and anti-migraine agents [26] may also contribute to salivary disorders. The second main cause are diseases like Sjogren’s syndrome [27,28], diabetes [29,30,31], depression [32,33], anemia [34], bulimia [35] and genetic disorders (i.e., Down syndrome [36], Prader–Willi syndrome [37]). Problems with the dry mouth were also observed in alcoholics [38], cigarette smokers [39] and drug addicts [40,41,42,43]. The third main cause is radiation therapy of patients that develop cancer in head and neck area [44,45,46,47]. Irradiation causes degeneration of the salivary glands tissue causing reduction of saliva secretion. However, patient response to radiotherapy is individual and depends on the radiation dose and treatment area. In effect, the application of this therapy might provide to the short-term dryness or leads to a complete lack of saliva production.

Another factor associated with reduced salivation is aging. Research carried out in different age groups, clearly indicate the prevalence of problems with the secretion of saliva in elderly people [48,49]. However, it correlates with the more frequent taking of medicines due to the occurrence of diseases. On the other hand, some authors [50,51] indicate a lack of significant differences in salivary secretion between young and elderly (both healthy and non-medicated) people. On the other hand, it is known that the composition of saliva changes in the elderly age—especially the differences were observed with regards to the level of sodium and potassium ions, proline-rich proteins, IgA, lactoferrin, and lysozyme [52,53]. In addition, some drugs such as anticholinergics cause more salivary problems in the elderly than in young people [50]. Similar observations are reported in the case of the influence of diseases on the secretion of saliva among people of all ages [51]. Considering all these factors, the problem with salivary secretion in the elderly is a fact. Xerostomia in the elderly is usually more severe due to less regenerative abilities, missing teeth and the need to use dentures.

Qualitative changes in saliva may not only show some of the inefficiencies of the salivary glands but also provide diagnostic potential. Recently published studies [54,55] established that saliva has been useful as a liquid biopsy for the diagnosis of various oral or systemic diseases, including cancer. As suggested in the article of Khan et al. [56] salivary based diagnostics is a developing field to achieve the level of point-of-care technology, in identification and validation of biomarkers via application of toolboxes and other class of devices for the early detection and diagnosis of several oral and systemic diseases in a non-invasive, easily-monitored, less time consuming, and in a personalized manners. However, this diagnostic process might be impeded in patients with xerostomia syndrome.

## 3. Therapeutic Options

**Figure 2:**
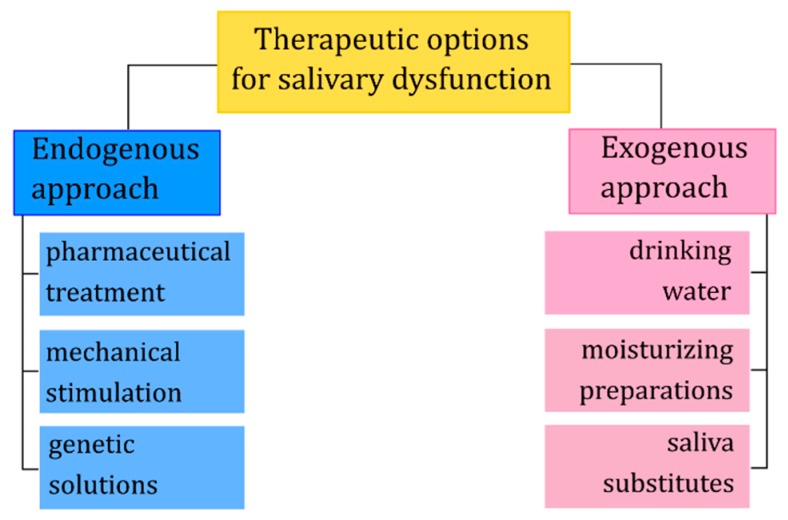
Therapeutic options for salivary dysfunction.

Depending on the degree of salivary dysfunction, there are different therapeutic methods to restore the lost functions, alleviating symptoms, preventing and correcting the possible consequences of the lack of natural saliva. Generally, these approaches can be divided into endogenous and exogenous (Figure 2). The endogenous approach involves replace or enhancement of salivary glands function through pharmaceutical or genetic modifications. Typically, such modifications are intended to stimulate the secretion of water, electrolytes as well as macromolecules, or preventive protection against harmful factors such as ionizing radiation. The exogenous approach involves the topical application of saliva substitutes to replace lost or enhance existing function(s) of natural saliva [57].

### 3.1. Methods for Salivary Glands Stimulation or Protection

Among the endogenous pharmaceutical solutions, there are parasympathomimetics such as pilocarpine [58,59], pilocarpine combination with ANTT (anethole trithione) [60], cevimeline [61,62] and bethanechol [63,64]. These substances are muscarinic receptor agonists whose stimulation increases the secretion of saliva and they are used to relieve the symptoms of xerostomia induced by radiotherapy. However, pilocarpine may cause adverse cardiovascular and pulmonary effects. In studies on the efficacy of pilocarpine [65], for safety reasons, patients with the uncontrolled cardiac and pulmonary disease were excluded. Due to possible interactions, pilocarpine is not recommended in patients with xerostomia induced by medication such as beta-blockers, anticholinergics, antidepressants or antihistamines [66]. What more, their action may cause nausea and dizziness. Other drugs that increase the salivation are bromexine [67] and nizatidine [68]. 

The chemical composition of oral fluid samples depends on many factors, as i.e.: stimulation rate or type of saliva collectors used during saliva collection. Lomonaco et al. [69] showed that i.e., urate and lactate concentrations in oral fluid decrease with the increase of the stimulation and oral fluid flow rate. Nevertheless, it progressively increases at higher stimulations. Also, the method of saliva flow stimulation (unstimulated, mechanical or chemical stimulation) influences on the level of total protein, CRP and IgE concentrations. At work of Groschl and Rauh [70] the reliability of commercial saliva collectors for the analysis of salivary hormones was analyzed. Based on their experimental results, the authors recommend the type of device for saliva steroid analysis. Mechanical stimulation of salivation is mainly achieving by chewing gum without sugar (containing xylitol and sorbitol with antimicrobial effect). Other methods include electrostimulation [71], acupuncture [72] and the positive effect of hyperbaric chambers [73,74].

Irradiation of the salivary glands during radiotherapy is associated with permanent damage to the cells and inability to secrete saliva. The protection of irradiated cells (preventing the formation of free radicals and protecting DNA molecules) can be provided by cytoprotective drugs [75], and in the future, gene therapies. One of these cytoprotective drugs is amifostine, which mechanism of cell protection occurs by scavenging oxygen-free radicals and donating hydrogen to repair damaged target molecules. Studies have shown that this allows to prevent acute xerostomia and inhibit the development of late xerostomia [76,77]. Gene therapies are not well known in clinical practice, but there are studies [78,79,80] showing their potential use in the protection of salivary glands against the harmful effects of ionizing radiation. In preclinical animal studies (where the model animals were miniature pigs weighing 30-40 kg) [78], a replication-deficient, recombinant adenovirus encoding human aquaporin-1 (hAQP1) was administered to the irradiated submandibular glands and a three-fold increase in salivary secretion was observed comparing to control-virus treated glands. Other potential solution use in gene therapy may be gallic acid. Palaniyandi et al. [79] functioning as a TLK1/B modulator that has antioxidant and free radical scavenging activity. Irradiated cells treated with gallic acid showed, among others, a better clonogenic survival in comparison to untreated controls. Research is also conducted [81] on growth factors responsible for apoptosis inhibition and increase proliferation of acinar cells after radiation. Administration of keratinocyte growth factor (DeltaN23-KGF) 4 days before irradiation (in mice), increased number of stem/progenitor cells and acinar cells survived after radiation, preventing hyposalivation.

### 3.2. Symptomatic Therapies: Saliva Substitutes

The exogenous approach is based on symptomatic therapy. Usually, when the symptoms of dry mouth are not significant, patients drink large quantities of water. However, the water itself is not able to provide adequate hydration and lubrication and does not provide antimicrobial properties. Saliva preparations are a better solution. Usually, these preparations are characterized by higher viscosity than water, similar to the viscosity of natural saliva. They should provide protection of tissues, facilitate speaking/eating and counteract the symptoms of xerostomia such as dental caries, remineralization of teeth or inflammation of the mucous membrane. 

Saliva substitutes may contain substances of natural origin (salivary macromolecules such as mucins, lysozyme, lactoferrin) that provide high biocompatibility. However, these are compositions mainly based on rheological modifiers [82,83] (xanthan and guar gums, carboxymethyl cellulose (CMC), glycerol), electrolytes, preservatives, and sweeteners. Many authors [84,85,86] analyzed literature data on clinical and laboratory tests of saliva substitutes. The results of the studies indicate that in patients with xerostomia (mainly after radiotherapy), commercially available saliva preparations seem to significantly reduce the symptoms of dry mouth. Mostly, however, these are oral lubricants, which replace the need for frequent drinking of water, moisturizing the mucous membrane and relieving discomfort in the mouth. In general, lubrication of the oral mucous membrane reduces the symptoms, although the effects are short-lived [87]. New types of salivary substitutes, often in the form of gels or mouthwashes, try to mimic some of the properties of human saliva and contain antimicrobial substances and have some buffering and re-mineralizing properties. Unfortunately, the data on the proper effectiveness of these preparations are ambiguous. Literature data have some risk of bias [85], studies are conducted on a small group of patients and contain subjective information. In general, according to previous results [88,89,90,91], mucin-based substitutes seem to be better than preparations based on carboxymethylcellulose due to rheological and lubricating properties (see Section 4.1). Some promising in vitro results were obtained for substitutes containing natural components such as lysozyme, hyaluronic acid or peroxidase [92]. However, there is no data on more complex substitutes that would have physicochemical, rheological and lubricating properties similar to saliva, containing antimicrobial components, and having immunomodulatory and remineralization properties.

## 4. Artificial Saliva

### 4.1. Rheological and Lubricating Properties of Artificial Saliva

One of the key issues in the development of artificial saliva is an appropriate adjustment of rheological characteristics to natural saliva. It allows providing lubricating properties that are crucial in the protection of tissues, proper functioning of the speech apparatus and food intake. In addition, it helps to reduce the discomfort in the mouth resulting from the presence of liquid behaving differently than natural saliva.

**Figure 3:**
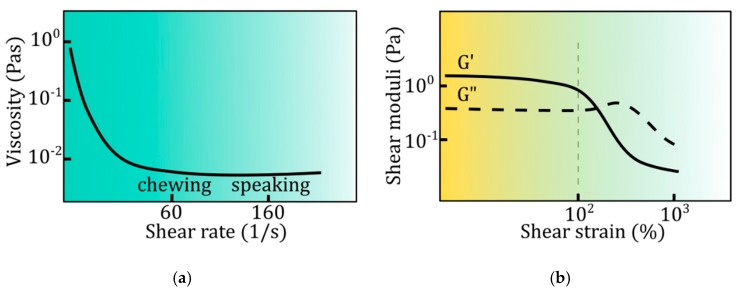
Rheological properties of human saliva: (**a**) viscosity in shear rate function, (**b**) shear moduli (G’ is an elastic/storage modulus, G” is viscous/loss modulus) in shear strain function.

Natural saliva is a non-Newtonian fluid. This means that its viscosity (a measure of the internal friction resistance) varies depending on the shear rate (Figure 3a). The viscosity of resting saliva, when the shear rate is in the range of 0.1–1 1/s, is much higher than the viscosity during chewing and speaking when the shear rate is about 60 and 160 1/s [93]. This dependence of viscosity on the rate of deformation is called pseudoplasticity and is important for proper functioning. Substitutes based on carboxymethylcellulose or glycerol are Newtonian fluids, which viscosity is usually higher comparing to natural saliva. Closer rheological characteristics to natural saliva have substitutes containing mucin or mucin with xanthan or guar gum [88,89,90].

Another rheological characteristic of saliva is viscoelasticity, which exhibits indirect behavior between a viscous fluid and an elastic solid. Viscoelasticity is tested in vitro using rheometers under creep/recovery or dynamic tests (by means of forced oscillations). In the conditions of small deformations, saliva behave more elastic than in situations where the deformations are significant, for example during the speaking process (Figure 3b). In fact, it is a manifestation of a complex structure of saliva, which is not a normal solution, but rather a weak gel [94]. Substitutes based on polysaccharides like xanthan gum are characterized by different viscoelastic properties comparing to natural saliva. For example, taking into account the van der Reijden studies [89], the ratio of viscous to elastic part (η′/η″) (for low shear rates < 1.5 1/s) for natural saliva, is 2.4, for xanthan gum 3.72, for CMC 17, for hydroxyethylcellulose 33.3. Porcine gastric mucins, used as a substitute for salivary mucins, have very low viscoelastic properties. However, besides the viscoelastic properties of the lubricating layer, the role of mucoadhesive properties is crucial. Therefore, mucin-based formulations containing rheological modifiers in the form of polysaccharides, such as xanthan gum, can counteract the properties of natural saliva [95]. 

As mentioned earlier, rheological properties, such as viscosity and viscoelasticity, are important for lubrication processes. Lubrication has been defined as the ability of a substance to reduce friction between two moving surfaces and is a major function of saliva in the oral cavity. However, there is a conviction in a strong correlation between the high viscosity of the lubricant and the reduction of friction (manifested, for example, in a decrease in friction coefficient). In practice, depending on the conditions under which the tests are carried out, obtained results differ from each other, with little correlation with viscosity. In studies [90,96], saliva and mucin-based saliva preparations, despite lower viscosity, showed better lubricating properties than high viscosity substitutes based on carboxymethylcellulose or glycerol. On the other hand, Reeh et al. [97] in their research showed similar lubricating properties of mucin and/or CMC-based substitutes, however, they were lower than the glycerol- and SDS-based substitutes. The relationship between rheological properties and lubrication is more complicated. It is necessary to distinguish between hydrodynamic lubrication, in which the friction surfaces are completely separated by a layer of lubricant, from boundary lubrication, in which the surfaces may occur in direct contact with each other. The viscosity of the lubricant is important in the hydrodynamic regime but plays a minor role in the boundary lubrication, where layer adsorbed on the oral surfaces are very important in the context of lubrication. Thus, depending on the operating conditions of the friction system, a lubricant with different properties is needed. The exact conditions of friction in the oral cavity are not known. However, it can be suspected, as in the case of synovial fluid, nature has equipped us with the best tools [98,99]. Rheological properties of natural saliva change depending on the degree and rate of deformation. Therefore, when developing artificial saliva, we should strive to imitate its rheological behavior.

It is important to know that rheological tests are in vitro and it is difficult to determine how the test conditions reflect the natural ones and whether they are useful for estimating the effectiveness of substitutes in clinical applications.

### 4.2. Antimicrobial Properties of Artificial Saliva

**Figure 4:**
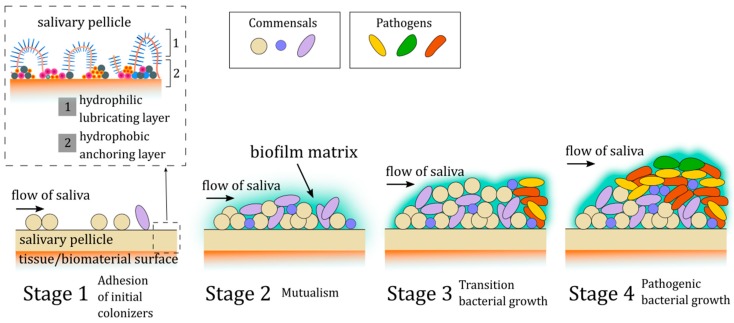
Diagram of adhesion and biofilm growth (based on [108]).

It is established that in the oral cavity, more than 700 bacterial species have been identified by culture and over 30% of them have been named. In effect, in each ml of saliva, around 108 CFU/mL can be detected and might be classified to eight different phyla including *Firmicutes, Actinobacteria, Proteobacteria, Fusobacteria, Bacteroides, Spirochaetes, Synergistetes*, and TM7x. Apart from the bacterial community, the oral microbiota is home for ultra-small bacteria (CPR; candidate phyla radiation group), as well as fungi and viruses [100]. However, in some clinical conditions, a dynamic shift in the oral microbiome with serious oral health consequences might take place. Due to this fact, restoring antimicrobial properties in artificial saliva preparation is crucial for its application in patients diagnosed with xerostomia. Hyposalivation generates an increase of bacteria that usually adopt a biofilm pattern of growth. These microbial changes might initiate other side effects that are associated with pH decreases. In effect, the consequences of xerostomia such as dental caries, gingivitis, oral candidiasis, halitosis, and periodontal disease, might become a serious oral health issue. The above was confirmed by a previously published study [101], where results demonstrated the relevance of etiology of xerostomia to the condition of the microflora of the oral cavity. The authors show the impact of hyposalivation on higher counts of *Lactobacillus* spp. and revealed an association between duration of xerostomia, and high counts of *Candida* spp. and *Lactobacillus* spp. In turn, the results presented by De Ryck et al. [102] supported the hypothesis that interruption of the salivary flow and associated xerostomia following radiation therapy were linked to shifts in the oral microbiome causing higher abundances of *Candida* spp. (*C. albicans*), *Staphylococcus* spp. and cariogenic species. In effect, this observation might explain the higher prevalence of candidiasis and caries in patients treated with radiotherapy, while decreasing the number of *S. sanguis*, *Fusobacterium* spp. and *Neisseria* spp. Moreover, in patients during radiotherapy for nasopharyngeal carcinoma observed dysbiosis of oral mucosal microbiota might lead to exacerbating the severity of mucositis [103]. Khovidhunki et al. [104] observed that patients with diabetes mellitus (DM) diagnosed with hyposalivation were characterized by a higher number of *Lactobacilli* spp. and *Candida* spp. and mutans *Streptococci* in the saliva compared with those without dry mouth symptoms. This result suggests a strong association between xerostomia and alterations in oral microbiota in patients with DM. Additionally, as a result of a shift in oral microflora and disorder of salivary protection mechanisms, in the case of ventilated patients at intensive care units, accumulation of dental plaque and candidiasis of oral mucosa might lead to developing of lung infections. To reduce the risk of lung infections in these patients, oral hygiene in combination with oral antiseptics are recommended [8]. However, the application of these procedures does not resolve all problems that are associated with the hyposalivation state. In accordance to this the ability of three different commercially available artificial saliva preparations was evaluated to reduce adhesion of different oral pathogens including *Streptococcus mutans* alone and in co-culture which representative Gram-negative bacteria such as *E. coli*, *P. aeruginosa* and Gram-positive strains including *S. aureus* and fungi (*Candida* spp.). Tested strains were isolated from oncological patients with immunology disorder from the oral cavity, which are in a group of higher preference to develop hyposalivation and xerostomia. All evaluated preparations contain xylitol, while preparation B and C possess additionally plant or herbal extract. Dental plaque is a mixed-species biofilm composed of more than 500 bacterial species that accumulates on the teeth. The colonization process follows a specific pattern with the first step - adhesion of initial colonizers to the enamel salivary pellicle, and then secondary colonization via interactions occurring between bacteria [105,106]. Interbacterial adhesion is mediated by components of the plaque matrix allowing the microorganisms to accumulate and to provide cohesive properties [107] (Figure 4). Since these interactions contribute to dental plaque development and finally different diseases such as caries and periodontal disease, reduction/inhibition of this stage is crucial and is an urgent need in prevention therapy.

**Figure 5:**
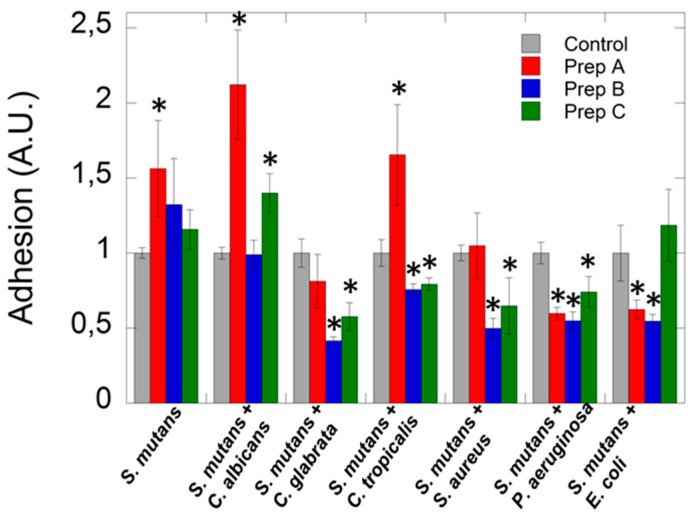
The impact of commercially available artificial saliva preparations on microorganism adhesion. The graph compared the abilities of tested microorganisms to adhere to wells of polystyrene microtiter plates in the presence of artificial saliva preparations using an adhesion assay that based on CV-staining method.

As presented in Figure 5 commercially available artificial saliva preparations does not exert any effect to restrict the adhesion of *Streptococcus mutans* during 3 h incubation. In the case of fungal-based mixed species co-culture, a different influence was observed, which probably depends on species of *Candida*. Lack of reduction or increased adhesion has been observed if *Candida albicans* with Streptococcus mutans was cultured. While in the case of co-culture of *C. tropicalis* or *C. glabrata* with *mutans Streptococci*, inhibition of adhesion was established at 20% and 40 % levels, respectively, compared to control after addition preparation B or C. In the case of co-culture of representative of Gram-positive bacteria with *S. mutans*, we observed reduction of adhesion up to 50 % after addition preparation B or C, which have an ingredient of plant or herbal origin, compared to preparation A the addition of which does not affect microorganism adherence. In turn, incubation of preparation A or B with a combination of Gram-negative representatives of bacteria with *S. mutans* caused ~45% restriction of microorganism adhesion to the surface in comparison to control and formulation C. Therefore, there is a real need for products which will possess antimicrobial properties and will regulate the oral microbiota and reduce the risk of oral infections in patients with hyposalivation.

### 4.3. Development of Artificial Saliva

Research in the field of development of artificial saliva that reproduces the properties of natural saliva requires knowledge of the structural–functional relationships of individual salivary molecules. Although 99.5% of saliva is water, these molecules form a complex structure. Glantz [109] proposed a four-level structural model of saliva consisting of a liquid phase of the electrolyte solution, a scaffold-like continuous network structure, less water-soluble proteins, and other salivary molecules suspended in the network structure, and phase of microbial and epithelial cells.

The largest molecules of saliva, and at the same time the most responsible for its properties, are mucins MG2 (130 kDa) and MG1(>1000 kDa) [110,111,112]. Their interaction with other, smaller particles provides saliva with unique properties. For example, the creation of a film that protects tissues is related to forming a hierarchical structure of high molecular weight, highly glycolyzed and hydrated mucins, the layer of which is strengthened by smaller proteins [113,114] (Figure 4). Adsorbed proteins and glycoproteins are not only able to provide mechanical protection by creating a lubricating layer, but also this form allows agglomeration of microorganisms [107,115,116].

Levine [57] distinguished five key features of saliva molecules. First of all, the shape and conformation of particles are important for fulfilling biological functions. For example, statherin and histatin achieve the highest biological activity when they adopt the α-helical conformation. Amylase loses its properties (digestion of starch, binding with hydroxyapatite) when its disulfide bonds are damaged, and the superstructure of the molecule is disturbed. The second key feature is the multifunctionality of saliva molecules. Larger particles, such as mucins, are responsible for covering, protecting and lubricating of oral tissues. They participate in the formation of the food bolus. They also have the ability to interact with bacteria, fungi, and viruses. Smaller particles are responsible for mineralization, buffering and support lubrication in the oral cavity. Importantly, the individual functions of the particles overlap, so that the deficiency of one component of saliva can be compensated by the presence of the other—this is the third feature of saliva particles. The fourth feature is amphifunctionality. It manifests itself in the different behavior of some particles, depending on the place where they are (as well as the involvement of another fragment of the molecule). The best example is amylase, which in the solution is able to bind bacteria and facilitate their removal. Adsorbed to the surface, by digesting maltose starch, it provides food products to the bacteria that bind to them, which secrete harmful acids inducing enamel digestion.

The development of artificial saliva is associated with the use of molecules that will replace the functions of saliva molecules. They may be of animal origin and such solutions are already known, e.g., replacement of salivary mucin with mucin derived from pigs or cows [91,112,117]. These molecules can also be synthesized in the laboratory. 

Levine [57] divided saliva preparations into two categories. The first includes artificial saliva containing molecules obtained by recombinant methods similar to human molecules. While it is possible to reconstruct the structure of small particles such as histatin or statherin, it is extremely difficult to obtain a mucin structure in a proper conformation. 

The second type relates to recombinant particles that contain improved bioactive domains or domains that are characteristic of various proteins. In effect, multifunctional molecules are obtained. One of these solutions is the application of drug delivery systems containing metallic nanocarriers, for example, silver, gold or iron oxide magnetic nanoparticles (MNP) [118,119]. The above-listed nanoparticles possess proved antimicrobial activity, however, among them, MNP offers a great promise in the development of modern medical applications. This is strongly associated with their unique magnetic properties that provide the opportunity to create targeted drug delivery via using an external magnetic field. In the case of infection, these features will permit us to increase their in situ local concentration and enhance their effectiveness. Moreover, these specific features might be applied both in vitro and in vivo setting as diagnostic tools or therapeutic/imaging agents [120]. In effect, in the case of oral infections that are a characterized as a heterogeneous group of diseases, the recognition of a theranostic approach via combined real-time diagnostics and observation of the treatment progress are the main purpose in nanomedicine. Moreover, a lot of studies indicated that some other properties of magnetic nanoparticles such as resistance to biodegradation processes, surface activity and ability to penetrate bacteria cell membranes, might be useful to develop new antibacterial treatments [121]. Based on our [122] and previously published results [123], the proposed mechanism of antibacterial action of magnetic nanostructure involves their contribution to the generation of reactive oxygen species (ROS), inactivation the specific proteins, destruction of the cell membrane and interference with bacterial electron transport of oxidation of NADH. Additionally, Park et al. [124] showed that magnetic nanoparticles have the capability to penetrate the biofilm and are able to inactivate of microbial cells in the presence of an external magnetic field, especially after induction of hyperthermia. Furthermore, functionalization of their surfaces provides the opportunity to attach active molecules via physicochemical interaction or covalent bonding to create a drug delivery system. These molecules can belong to a different group of active agents such as antiseptics used in dentistry as chlorhexidine [125], or antibacterials including antibiotics, chemotherapeutic or peptides and their analogs [126,127]. Numerous studies of core-shell nanostructures (containing antibacterial peptides and their analogs, ceragenins) [128,129] showed high antibacterial and antifungal activity in relation to drug-resistant microbial strains while maintaining high biocompatibility. Importantly, it is established that the presence of nanoparticles as drug carriers might modulate and expand the spectrum of action of classical antimicrobial agents. They are able to interact with antimicrobial agents via synergistic or additive manner as well as increase their killing-activity in the media mimicking site of infection [129,130]. Those results indicated that the use of nanotechnology has a very high potential, but there are concerns regarding the high reactivity of nanoparticles including activation of the immune system and interaction with other active molecules in the human body. Due to this, more in vivo studies are needed to develop nano-formulations that might be administered via intravenous or topical application to treat local or systemic infections as well as to cover medical devices to protect before the formation of the biofilm. However, it is generally accepted that nanoparticles with a hydrodynamic diameter of 10–100 nm, low size- and shape-polydispersity and adequate surface functionalization by polymers and/or homing molecules possess optimal pharmacokinetic properties for in vivo applications [131,132].

## 5. Conclusions

Saliva is crucial for the health and proper functioning of the oral cavity. Dilution of its quantity or quality is associated with complications that increase suffering and lead to many diseases. Despite many therapeutic options, such as stimulation or protection of the salivary glands, the topical replacement of saliva by a substitute seems to be the only effective solution. The substitutes available on the market differ in properties from natural saliva and cannot replace it, hence, their low popularity. That is why it is important to develop artificial saliva that will mimic the complex properties of natural saliva.

Research in the field of development of artificial saliva should focuses on two groups of patients. The first are patients with salivary gland dysfunction, that is accompanied by a decrease in salivary secretion. In such cases, the saliva preparation should be as close as possible to natural saliva and reproduce its functions. The second are patients with salivary gland dysfunction, however, without problems with salivary flow. The development of such preparations requires an individual approach to the patient. Artificial saliva should support the function of natural saliva, for example through extended antimicrobial activity in patients with caries and mucositis. For patients from mechanical damage of hard and soft tissues, preparations with appropriate lubricating properties are needed.
